# Age-Progressive and Gender-Dependent Bone Phenotype in Mice Lacking Both Ebf1 and Ebf2 in Prrx1-Expressing Mesenchymal Cells

**DOI:** 10.1007/s00223-022-00951-7

**Published:** 2022-02-08

**Authors:** Vappu Nieminen-Pihala, Petri Rummukainen, Fan Wang, Kati Tarkkonen, Kaisa K. Ivaska, Riku Kiviranta

**Affiliations:** 1 Institute of Biomedicine, University of Turku, Turku, Finland; 2 Present Address: Orion Pharma, Turku, Finland; 3 Division of Medicine, Department of Endocrinology, University of Turku and Turku University Hospital, Turku, Finland

**Keywords:** Ebf1, Ebf2, Osteoblast, Conditional knockout mouse model, Prrx1, Age

## Abstract

Ebfs are a family of transcription factors regulating the differentiation of multiple cell types of mesenchymal origin, including osteoblasts. Global deletion of Ebf1 results in increased bone formation and bone mass, while global loss of Ebf2 leads to enhanced bone resorption and decreased bone mass. Targeted deletion of Ebf1 in early committed osteoblasts leads to increased bone formation, whereas deletion in mature osteoblasts has no effect. To study the effects of Ebf2 specifically on long bone development, we created a limb bud mesenchyme targeted Ebf2 knockout mouse model by using paired related homeobox gene 1 (Prrx1) Cre. To investigate the possible interplay between Ebf1 and Ebf2, we deleted both Ebf1 and Ebf2 in the cells expressing Prrx1. Mice with Prrx1-targeted deletion of Ebf2 had a very mild bone phenotype. However, deletion of both Ebf1 and Ebf2 in mesenchymal lineage cells lead to significant, age progressive increase in bone volume. The phenotype was to some extent gender dependent, leading to an increase in both trabecular and cortical bone in females, while in males a mild cortical bone phenotype and a growth plate defect was observed. The phenotype was observed at both 6 and 12 weeks of age, but it was more pronounced in older female mice. Our data suggest that Ebfs modulate bone homeostasis and they are likely able to compensate for the lack of each other. The roles of Ebfs in bone formation appear to be complex and affected by multiple factors, such as age and gender.

**Supplementary Information:**

The online version contains supplementary material available at 10.1007/s00223-022-00951-7.

## Introduction

Early B-cell factors (Ebfs) are a four‐member family of transcription factors (Ebf1-Ebf4) that regulate the differentiation of e.g. neuronal cells [1, 2], B-cells [3, 4] and adipocytes [5, 6]. The role of Ebf1 is prominent in the white adipose tissue [7], whereas Ebf2 maintains brown adipocyte identity [8].

Ebf family members are all expressed in the bone marrow niche [9, 10], and Ebf1 and Ebf2 have been implicated in bone homeostasis. Global deletion of Ebf1 (Ebf1KO) leads to increased bone mass due to increased bone formation [11]. Conditional knockout models have suggested varying roles for Ebf1 in osteoblast differentiation. Zee et al. reported that targeting Ebf1 deletion to osteoblast lineage using Runx2-Cre had no effect on osteoblast differentiation or bone accrual [12]. In line with this, Seike et al. observed that targeting Ebf1 deletion to early limb bud mesenchyme with paired related homeobox gene 1 (Prrx1) did not lead to detectable bone abnormalities [9]. In contrast, Derecka et al. reported that Prrx1-driven deletion of Ebf1 leads to increased bone formation [10].

We have studied the effect of Ebf1 on bone metabolism using two different conditional knockout mouse models. Deletion of Ebf1 in the early-stage osteoblasts expressing Osterix (Osx) led to increased bone formation in vivo, whereas deletion in the mature osteoblasts expressing osteocalcin (OCN) had no effect [13]. We also identified a putative EBF1 binding site in the Osterix promoter by ChIP assay in MC3T3-E1 osteoblasts and showed that EBF1 was able to activate Osx-luciferase reporter construct that included the identified EBF1 binding site. Our data suggest that Ebf1 promotes early osteoblast differentiation by directly inducing Osterix expression, but it suppresses bone formation in differentiated mature osteoblasts [13].

In contrast to Ebf1, global deletion of Ebf2 leads to decreased bone mass due to enhanced bone resorption [14]. The expression of osteoprotegerin (OPG), an inhibitor of osteoclastogenesis, was also highly suppressed in the absence of Ebf2 [14]. More recently, downregulation of Ebf2 and subsequent decrease in OPG expression was linked to spinal cord injury induced osteoporosis in rats [15]. To the best of our knowledge, conditional knockout of Ebf2 in osteoblast lineage has not been reported.

These data suggest that Ebfs are capable of modulating bone homeostasis but the exact functions and molecular mechanisms of action at specific stages of osteoblast differentiation remain to be elucidated. They are likely to be able to compensate for the lack of each other, but the unique and redundant functions remain to be discovered.

We first examined the effect of Ebf2 downregulation using global knockout of Ebf2 on mice. These mice exhibited significant welfare issues with significant loss of body size and weight. To overcome these issues and to study the effects of Ebf2 specifically on long bone development, we created a limb bud mesenchyme targeted deletion of Ebf2 by using Prrx1-Cre. Unexpectedly, these mice had a very mild bone phenotype.

To further investigate the redundant and non-redundant effects of Ebfs on bone formation, we created Ebf1xEbf2_Prrx1_ mice in which both Ebf1 and Ebf2 have been deleted in cells expressing Prrx1. These mice had a pronounced bone phenotype which progressed with advanced age. For the first time, we also report a sexually dimorphic bone phenotype in relation to Ebf1 and Ebf2 deletion.

## Materials and Methods

### Experimental Animals

All mouse studies were approved by the Finnish ethical committee for experimental animals (license numbers 5186/04.10.07/2017 and 14044/2020), complying with the international guidelines on the care and use of laboratory animals, and were conducted under the supervision of the trained staff at the Central Animal Laboratory, University of Turku.

The targeting vector for Ebf2 was obtained from Soren Warming (Department of Molecular Biology, Genentech Inc., South San Francisco, CA, US). Chimera mouse line was generated by Turku Center for Disease Modeling (TCDM), University of Turku, Finland.

To generate global Ebf2 knockout mice, we first bred heterozygous Ebf2 chimeras with mice expressing Cre under the control of CMV enhancer—chicken-beta-actin (CAG) promoter. This led to deletion of exons 4 to 6 in all tissues of global Ebf2KO^+/−^ mice. As breeding Ebf2KO^+/−^ males and Ebf2KO^+/−^ females on C57BL/JC background led to unviable offspring, Ebf2KO^+/−^ were bred with 129SvJ mice. Breeding the following generation of Ebf2KO^+/−^ males and Ebf2KO^+/−^ females on crossbred C57BL/JC and 129SvJ background led to more viable global Ebf2KO^−/−^ offspring. Male mice were analysed at 3 weeks of age.

Conditional knockout mice generation started by first crossing heterozygous Ebf2 mice from the chimera breeding with Flippase (Flp) expressing mice to remove the Neo cassette flanked by Flp recombinase target sites (Frt) and create Ebf2^fl/+^ mice (Supplemental Fig. 1a). Tissue specific conditional knockout in early limb bud mesenchyme was subsequently achieved by crossing Ebf2^fl/+^ with mice expressing Cre recombinase driven by the Prrx1 promoter specifically expressed in early limb mesenchymal cells (Tg(Prrx‐cre)1cjt/J mice), acquired from The Jackson Laboratory (Bar Harbor, ME, USA) [16]. Ebf2_Prrx1_ male and female mice were analysed, at 6 and 12 weeks of age.

Conditional double knockout Ebf1xEbf2_Prrx1_ mouse was created by crossing Ebf2_Prrx1_Cre+ males with Ebf1^fl/fl^Cre- [13] females. Ebf1xEbf2_Prrx1_ male and female mice were analysed, at 6 and 12 weeks of age.

Genotyping of mice was carried out from DNA extracted from ear marks of two‐week‐old to three‐week‐old mice. Cre recombination in the long bones was confirmed from genomic DNA isolated from humerus using isopropanol precipitation method (Supplemental Fig. 1b–d, Supplemental Table 1).

### Microcomputed Tomography

For the microcomputed tomography (μCT) bones were stripped from soft tissues, and stored in 70% EtOH at +4 C.

Femurs, tibias and vertebras were scanned and analysed with SkyScan 1072 or SkyScan 1272 X-ray computed tomography (Bruker-microCT, Kontich, Belgium). A plastic holder was used to ensure immobilization and constant positioning of the sample. The parameters used for scanning (in air) were X-ray tube voltage 70 kV, tube current 142 μA. The scanning voxel resolution for tibia was 8.37 μm, for femur 5 μm and for vertebra 7 μm. The bones were rotated in 0.4°degree steps (total angle, 360°) and an internal 0.5 mm aluminium filter was applied for beam hardening. Cross-sectional images were reconstructed with NRecon 1.6.4.1 software (Bruker-microCT).

For the analysis of the trabecular bone in the tibia, a region of interest (ROI) excluding the cortical bone was defined 80 layers (670 μm) distally from the proximal growth plate, for a total of 120 layers (1004 μm). Cortical bone was analyzed beginning from 5022 μm distally from the growth plate, for a total of 100 layers (837 μm). To analyse the secondary ossification centre (SOC) area of the tibia, a ROI excluding the cortical bone was defined 10 layers (84 μm) proximally from the proximal growth plate and extending a total of 12 layers (100 μm).

For the analysis of the trabecular bone in the femur, a ROI excluding the cortical bone was defined 200 layers (1000 μm) proximally from the distal growth plate, for a total of 120 layers (600 μm). Cortical bone was analyzed beginning from 5500 μm proximally from the growth plate, for a total of 100 layers (500 μm). As the Ebf1xEbf2_Prrx1_ mice had significantly shorter tibia and femur compared to controls, this was taken into account during the μCT-analysis. We calculated the proportional difference in bone length between control and knockout mice, in males and females. The difference in bone length was then translated to the distance of the ROIs from the growth plate.

For the analysis of the trabecular bone in the fourth lumbar vertebrae, a ROI excluding the cortical bone was defined 30 layers (210 μm) proximally from the distal growth plate and extending a total of 100 layers (700 μm).

### Histology and Histomorphometry

For histological analyses, tibias were fixed in 3.7% formalin, decalcified in EDTA, embedded in paraffin, and cut into 4 μm sections. Sections were deparaffinized, rehydrated, and stained with haematoxylin & eosin (H&E) using standard procedures. Samples were imaged with PANNORAMIC 1000 slide scanner (3DHISTEC, Budapest, Hungary).

Number of adipocyte ‘ghosts’ was counted manually from the secondary ossification centre areas in tibial sections using 3DHISTEC CaseViewer programme. The area of the secondary ossification centre was outlined and measured (mm^2^) using ImageJ software. Number of adipocyte ‘ghosts’ was normalized to the area and reported as adipocyte number/mm^2^ (Supplemental Fig. 7b). Bone marrow adiposity was calculated from tibial sections, a region of interest (1 mm^2^) was outlined inside the marrow cavity, and adipocyte ‘ghosts’ were counted manually. Bone marrow adiposity was reported as adipocyte number/mm^2^.

For the histomorphometric analysis, mice were injected with 20 mg/kg of Calcein (C0875, Sigma) and 40 mg/kg of Demeclocycline (D6140, Sigma) nine and two days prior to sacrifice, respectively. Tibias were harvested and fixed in 3.7% formalin o/n and transferred to 70% ethanol for a minimum of 3 days. The fixed bones were dehydrated with acetone and embedded in methyl methacrylate. Undecalcified 4-µm-thick sections were cut with hard tissue microtome (RM2255, Leica, Germany), deplastified and stained with Von Kossa method using the standard protocol to visualize mineralized bone, with Toluidine blue for osteoblast measurements and for Tartrate-resistant acid phosphatase (TRACP) to identify osteoclasts. The slides were analysed using Osteomeasure-histomorphometry workstation (OsteomeasureXP 3.1.0.1, Osteometrics, USA). The analysed area was defined as 1.30 mm × 0.84 mm, starting 200 µm distally from the growth plate, excluding the cortical border areas with a 100 µm margin. Static parameters were measured from slides with toluidine blue and TRACP staining and dynamic parameters from unstained slides according to standardized protocol [17].

Sagittal sections of proximal tibia stained with H&E were used for growth plate analysis. The average thickness of one medial and two marginal lines of proliferative and hypertrophic zones in growth plate was measured by Image J software. Cell density was analysed by calculating average cell number in eight regions (each 100×100 µm) of proliferative zone and in 16–17 regions (each 50×50 µm) of hypertrophic zone.

### Analysis of Gene Expression

Total RNA from the femurs was isolated by first cleaning the bones from soft tissue, cutting off the distal and proximal ends of the bone, removing bone marrow by brief centrifugation and pulverizing the bones in liquid nitrogen, followed by isolation using TRIsure reagent (Bioline) and RNeasy Mini Kit (Qiagen) according to the manufacturer’s instructions. cDNA was prepared with Sensifast cDNA synthesis kit (BioLine). Quantitative real-time PCR (qPCR) for the expression of osteoblast-related genes (Osx, Runx2, Col1a and ALP) was performed using Dynamo Flash SYBR Green qPCR Kit (ThermoFisher). The samples were analysed with Bio-Rad CFX96 qPCR system. The data was normalized using beta-actin as an internal control and the results were analysed by ^ΔΔ^Ct-method. Primer sequences are presented in Supplemental table 2.

### Statistical Analysis

Results are presented as mean ± SD. Statistical analysis was performed by two-tailed Student’s t test with Bonferroni’s comparison or Benjamini-Hochberg Procedure. *P*-values<0.05 were considered statistically significant and are indicated with asterisks as *P < 0.05; **P < 0.01 and ***P < 0.001.

## Results

### Global Deletion of Ebf2 Leads to Impaired Bone Formation

To test the function of Ebf2 in osteoblast differentiation and function we generated global Ebf2^−/−^ knockout mice (Ebf2KO). As the global deletion of Ebf2 leads to progressive developmental defects [2], the mice were analysed at three weeks of age to minimize the suffering of the animals. Body weight (*P*=0.001) and tibial length (*P*=0.03) were significantly reduced in three-week-old Ebf2KO male mice compared to controls (Supplemental Fig. 2a).

μCT-analysis presented significantly reduced trabecular bone volume (BV/TV, *P*=0.04) and increased trabecular separation (Tb.Sp, *P* =0.03) in three-week-old Ebf2KO male mice. Cortical bone thickness (Cort.Th, *P* =0.014), periosteal perimeter (Ps.Pm, *P* =0.004), endocortical perimeter (Ec.Pm, *P* =0.017) and mean moment of inertia (MMI, *P* =0.005) were also significantly decreased in Ebf2KO male mice compared to controls. This is in line with previously published phenotype of global Ebf2 deletion [14].

### Limb Bud Mesenchyme Specific Deletion of Ebf2 Results in a Mild Bone Phenotype

**Fig. 1:**
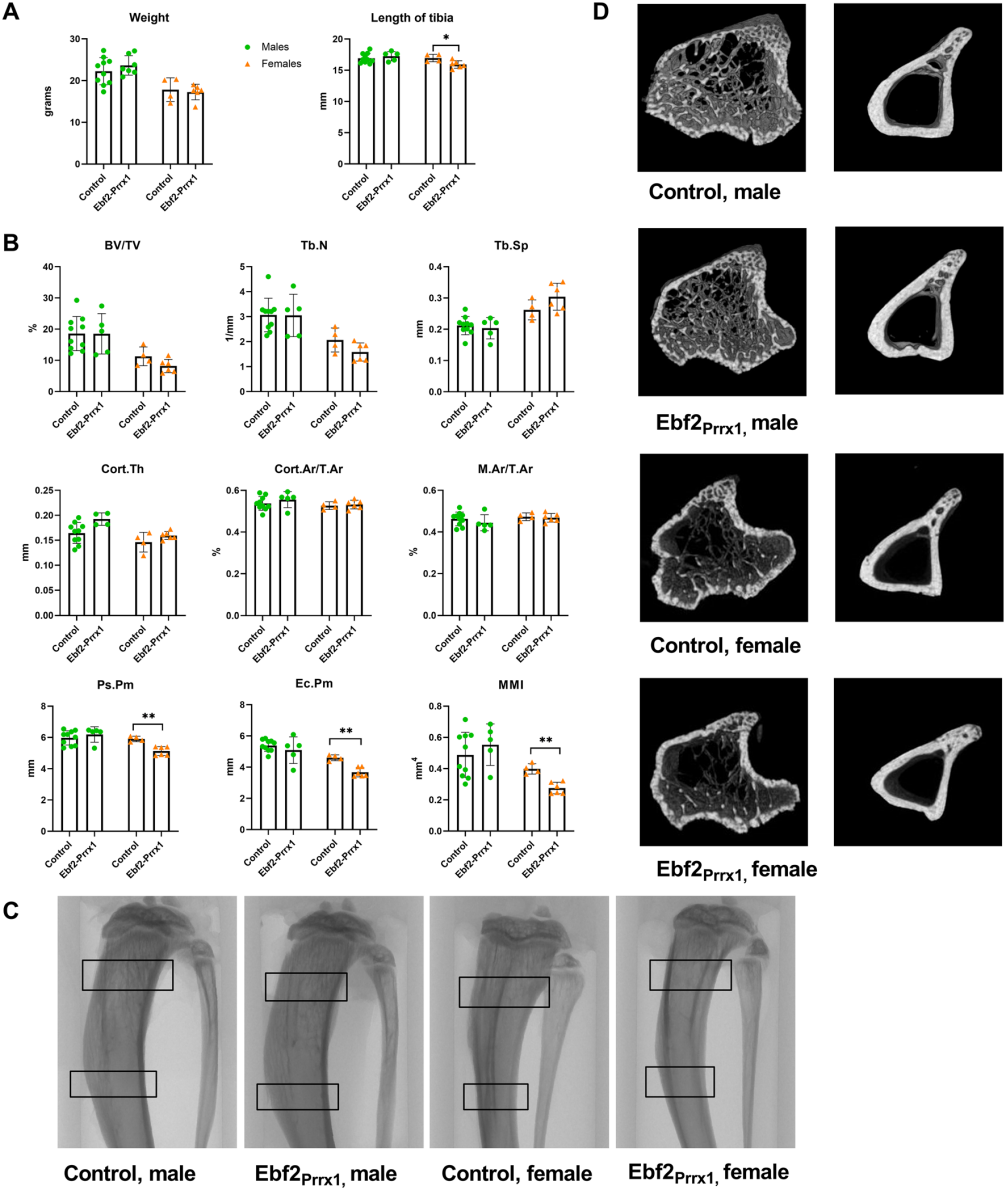
Tibial μCT-analysis of the six-week-old Ebf2_Prrx1_ mice. Ebf2_Prrx1_^−/−^ male mice (*n* = 5) had normal body weight and tibial length when compared to controls (*n* = 10). In six week old Ebf2_Prrx1_^−/−^ female mice (*n* = 6) tibial length was reduced when compared to controls (*n* = 4) (**A**). Results of the μCT-analysis were comparable between control and Ebf2_Prrx1_^−/−^ in males. Female mice were comparable to controls, except for significantly reduced periosteal perimeter, endocortical perimeter and mean moment of inertia (**B**). Representative coronal view of the tibia, trabecular ROI and cortical ROI are marked with black squares (**C**). 3D rendered representation of the trabecular and cortical bone region of interest in control and Ebf2_Prrx1_^−/−^ mice (**D**). Statistical significance was tested by two-tailed Student’s *t*-test with Bonferroni correction. *P*-values for significant differences between genotypes are presented. *P < 0.05; **P < 0.01

To evaluate the effect of Ebf2 knockout specifically on early long bone development, we created Ebf2_Prrx1_ mice in which Ebf2 deletion is driven by the limb bud mesenchyme specific Prrx1. Bone-targeted Cre recombination was verified by a PCR of genomic DNA from bone (Supplemental Fig. 1b). Mice were analysed at 6 and 12 weeks of age. At 6 weeks, body weight and tibial length were comparable between control and Ebf2_Prrx1_^−/−^ males (Fig. 1A). Body weights of the females were also comparable between control and Ebf2_Prrx1_^−/−^. However, tibial length was significantly reduced (*P*=0.03) in six-week-old Ebf2_Prrx1_^−/−^ females (Fig. 1A).

The bone phenotype was modest. In μCT-analysis six-week-old Ebf2_Prrx1_^−/−^ female mice were comparable to controls, except for significantly reduced periosteal perimeter (*P* =0.003), endocortical perimeter (*P* =0.001) and mean moment of inertia (*P* =0.004) (Fig. 1B). Six-week-old Ebf2_Prrx1_^−/−^ males were comparable to controls (Fig. 1B).

**Fig. 2:**
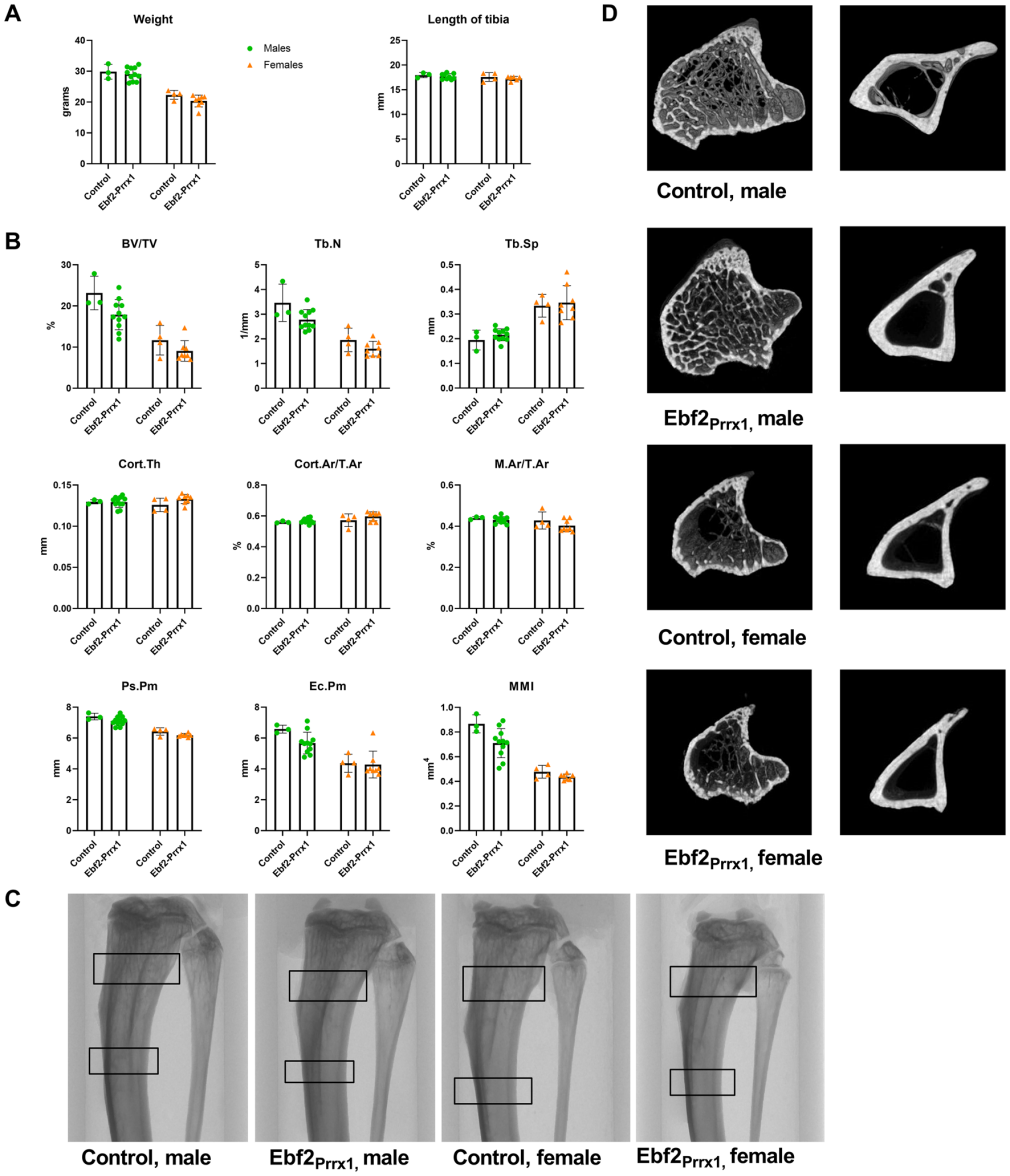
Tibial μCT-analysis of 12-week-old Ebf2_Prrx1_ mice. Ebf2_Prrx1_^−/−^ male mice (*n* = 11) had normal body weight and tibial length when compared to controls (*n* = 3). Effect on Ebf2_Prrx1_^−/−^ females (*n* = 8) compared to controls (*n* = 4) was the same (**A**). Results of the μCT-analysis were comparable between control and Ebf2_Prrx1_^−/−^ in male and female mice (**B**). Representative coronal view of the tibia, trabecular ROI and cortical ROI are marked with black squares (**C**). 3D rendered representation of the trabecular and cortical bone region of interest in control and Ebf2_Prrx1_^−/−^ mice (**D**). Statistical significance was tested by two-tailed Student’s *t*-test with Bonferroni correction

At 12 weeks of age there were no differences in weight or length of tibia between control and Ebf2_Prrx1_^−/−^ in males or in females (Fig. 2A). μCT-parameters were also comparable between controls and Ebf2_Prrx1_^−/−^, both in males and in females (Fig. 2B).

### Age-Progressive Decrease in the Length of Long Bones in Double Knockout Ebf1xEbf2_Prrx1_

To analyse the possible interplay between Ebf1 and Ebf2, we created a double knockout mouse model in which both Ebf1 and Ebf2 were deleted in cells expressing Prrx1 (E1E2-Prrx1). Bone-targeted Cre recombination was verified by a PCR using genomic DNA (Supplemental Fig. 1, c&d). Resulting Ebf1xEbf2_Prrx1_ mice were analysed at 6 and 12 weeks of age, in males and females.

**Fig. 3:**
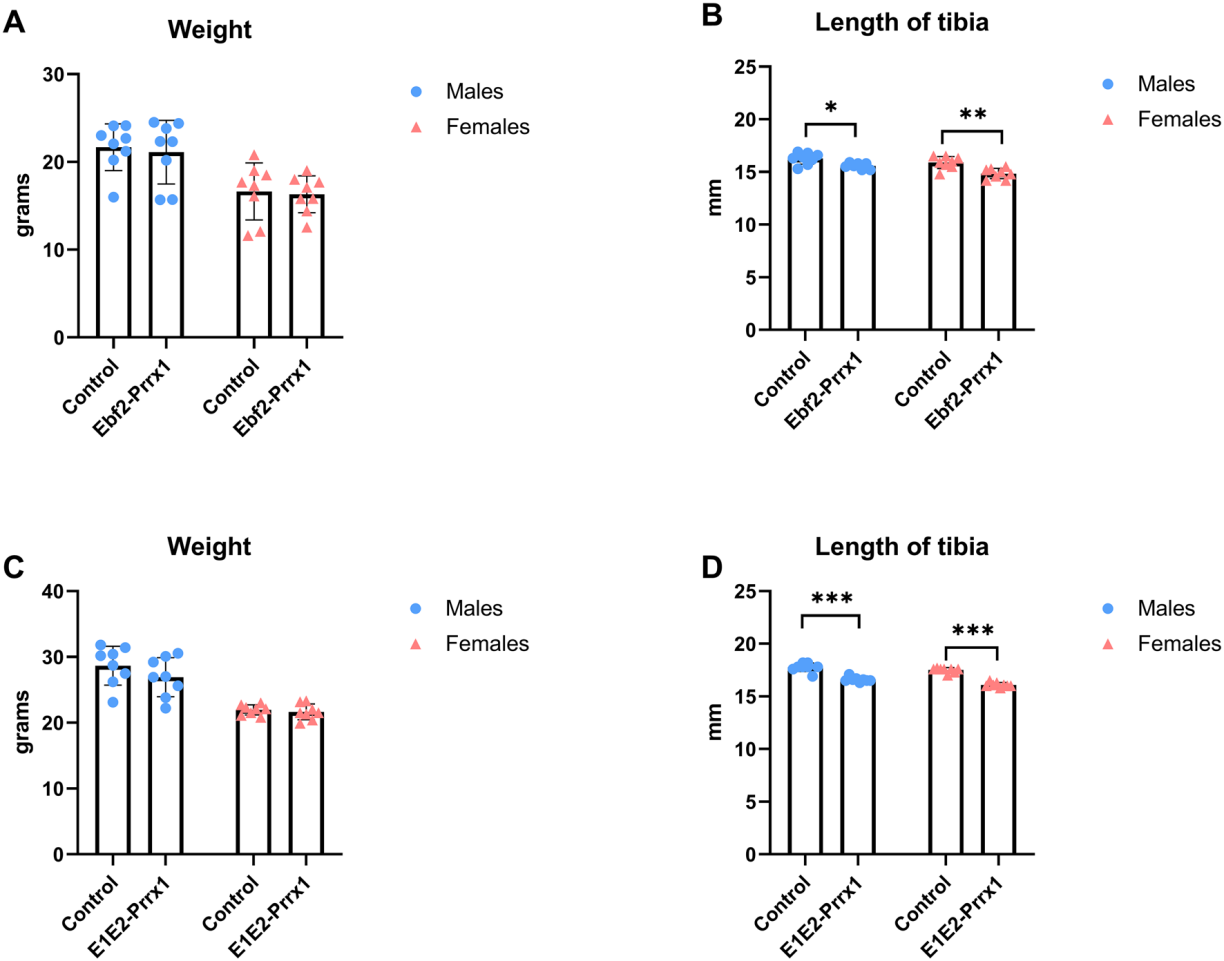
Weights and tibial lengths of six-week-old Ebf1xEbf2_Prrx1_ and 12-week-old Ebf1xEbf2_Prrx1_ mice (E1E2-Prrx1). Six-week-old Ebf1xEbf2_Prrx1_^−/−^ male (*n* = 8) and female (*n* = 8) mice had normal body weight (**A**) but significantly reduced tibial length (**B**) when compared to control males (*n* = 7) and females (*n* = 8). 12-week-old Ebf1xEbf2_Prrx1_^−/−^ male (*n* = 8) and female (*n* = 8) mice also had normal body weight (**C**), but even more pronounced reduction in tibial length (**D**) when compared to control males (*n* = 8) and females (*n* = 8). Statistical significance was tested by two-tailed Student’s *t*-test. *P*-values for significant differences between genotypes are presented. *P* < 0.05; **P < 0.01; ***P < 0.001

Body weight of male and female Ebf1xEbf2_Prrx1_^−/−^ mice were comparable to controls at both time points (Fig. 3A, C). Length of tibia was significantly reduced in Ebf1xEbf2_Prrx1_^−/−^ male (*P* = 0.015) and female mice (*P* =0.001) at 6 weeks (Fig. 3B). The difference in the length of tibia was even more pronounced in the later time point of 12 weeks in males (*P* <0.001) and in females (*P* <0.001) (Fig. 3D).

### Increased Bone Volume in Ebf1xEbf2_Prrx1_ Females

**Fig. 4:**
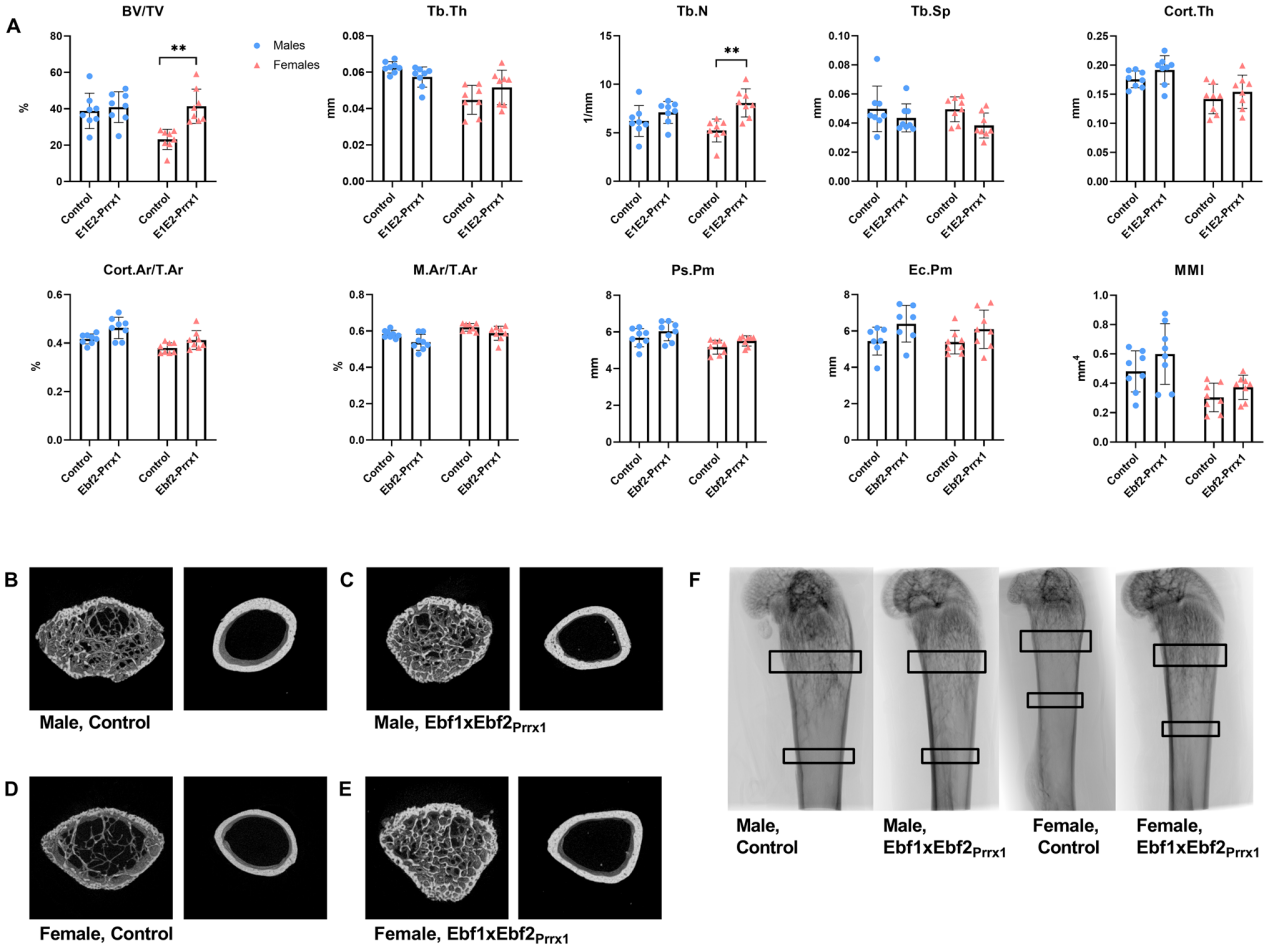
μCT-analysis of six-week-old Ebf1xEbf2_Prrx1_ mice femurs. Trabecular and cortical bone analysis revealed significant differences in Ebf1xEbf2_Prrx1_^−/−^ females (*n* = 8) compared to controls (*n* = 8) (**A**). Ebf1xEbf2_Prrx1_^−/−^ males (*n* = 8) were comparable to controls (*n* = 7) both in trabecular and cortical bone parameters (**A**). 3D rendered representation of the trabecular and cortical bone region of interest in control male (**B**), Ebf1xEbf2_Prrx1_^−/−^ male (**C**), control female (**D**) and Ebf1xEbf2_Prrx1_^−/−^ female (**E**). Representative coronal view of the femur, trabecular ROI and cortical ROI are marked with black squares (**F**). Statistical significance was tested by two-tailed Student’s *t*-test with Bonferroni correction. *P*-values for significant differences between genotypes are presented. *P < 0.05; **P < 0.01

We next analysed the bone phenotype of the Ebf1xEbf2_Prrx1_ mice by μCT-analysis. In six-week-old Ebf1xEbf2_Prrx1_^−/−^ females trabecular bone volume (*P* = 0.001) and trabecular number (*P* = 0.003) were significantly higher compared to controls in femurs (Fig. 4A). In tibia, the differences were less pronounced, cortical bone fraction was significantly increased (Cort.Ar/T.Ar, *P* = 0.04) whereas marrow area fraction was decreased (M.Ar/T.Ar, *P* = 0.04) (Supplemental Fig. 3). In six-week-old Ebf1xEbf2_Prrx1_^−/−^ male mice there were no significant differences in any of the femoral (Fig. 4A) bone parameters. In tibia marrow area fraction (*P* = 0.01), endocortical perimeter (*P* = 0.03) and mean moment of inertia (*P* = 0.03) were decreased in Ebf1xEbf2_Prrx1_^−/−^ males at 6 weeks of age (Supplemental Fig. 3a).

**Fig. 5:**
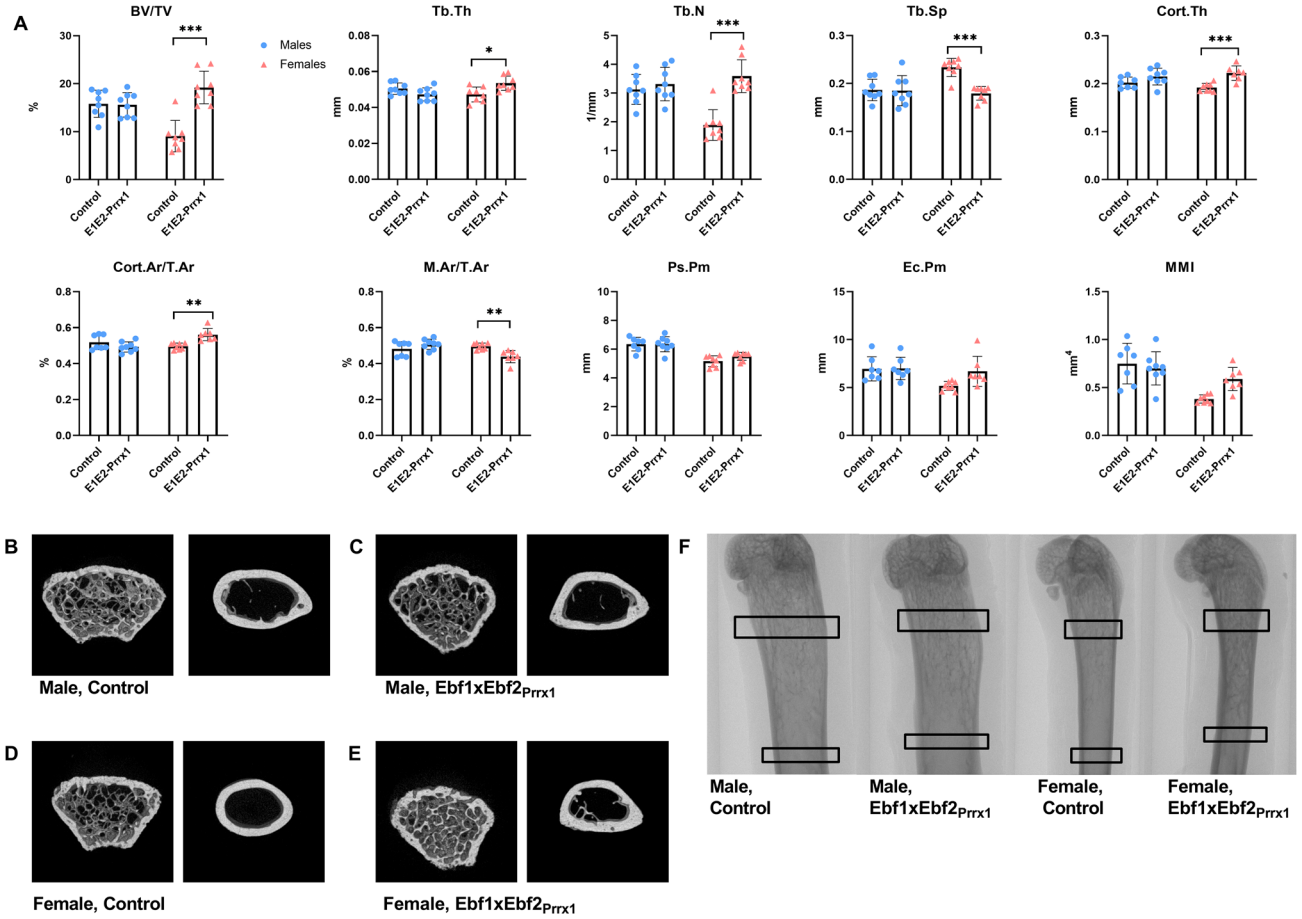
μCT-analysis of 12-week-old Ebf1xEbf2_Prrx1_ mice femurs. Trabecular and cortical bone analysis demonstrated significant differences in Ebf1xEbf2_Prrx1_^−/−^ females (*n* = 8) compared to controls (*n* = 8) (**A**). Ebf1xEbf2_Prrx1_^−/−^ males (*n* = 8) were comparable to controls *(n* = 8) both in trabecular and cortical bone parameters (**A**). 3D rendered representation of the trabecular and cortical bone region of interest in control male (**B**), Ebf1xEbf2_Prrx1_^−/−^ male (**C**), control female (**D**) and Ebf1xEbf2_Prrx1_^−/−^ female (**E**). Representative coronal view of the femur, trabecular ROI and cortical ROI are marked with black squares (**F**). Statistical significance was tested by two-tailed Student’s *t*-test with Bonferroni correction. *P*-values for significant differences between genotypes are presented. *P < 0.05; ***P < 0.001

The bone phenotype was more pronounced at the later timepoint of 12 weeks of age. In Ebf1xEbf2_Prrx1_^−/−^ females trabecular bone volume (*P* < 0.001), trabecular bone thickness (*P* = 0.03) and trabecular number (*P* < 0.001) were significantly increased in femurs. Accordingly, trabecular separation was significantly decreased (*P* < 0.001) (Fig. 5A). Cortical bone thickness was also significantly increased (*P* < 0.001), as well as cortical bone fraction (*P* < 0.001). Marrow area fraction was significantly reduced (Fig. 5A). Similar trend was observed in tibias (Supplemental Fig. 3b). We also analysed vertebrae from 12-week-old Ebf1xEbf2_Prrx1_^−/−^ female mice and they were comparable to controls, suggesting that the high bone mass is due to local, and not humoral mechanisms (Supplemental Fig. 4). 12-week-old Ebf1xEbf2_Prrx1_^−/−^ males had significantly lower trabecular thickness (*P* = 0.003) in tibias when compared to controls (Supplemental Fig. 3b). No differences were observed in the femurs (Fig. 5A).

**Fig. 6:**
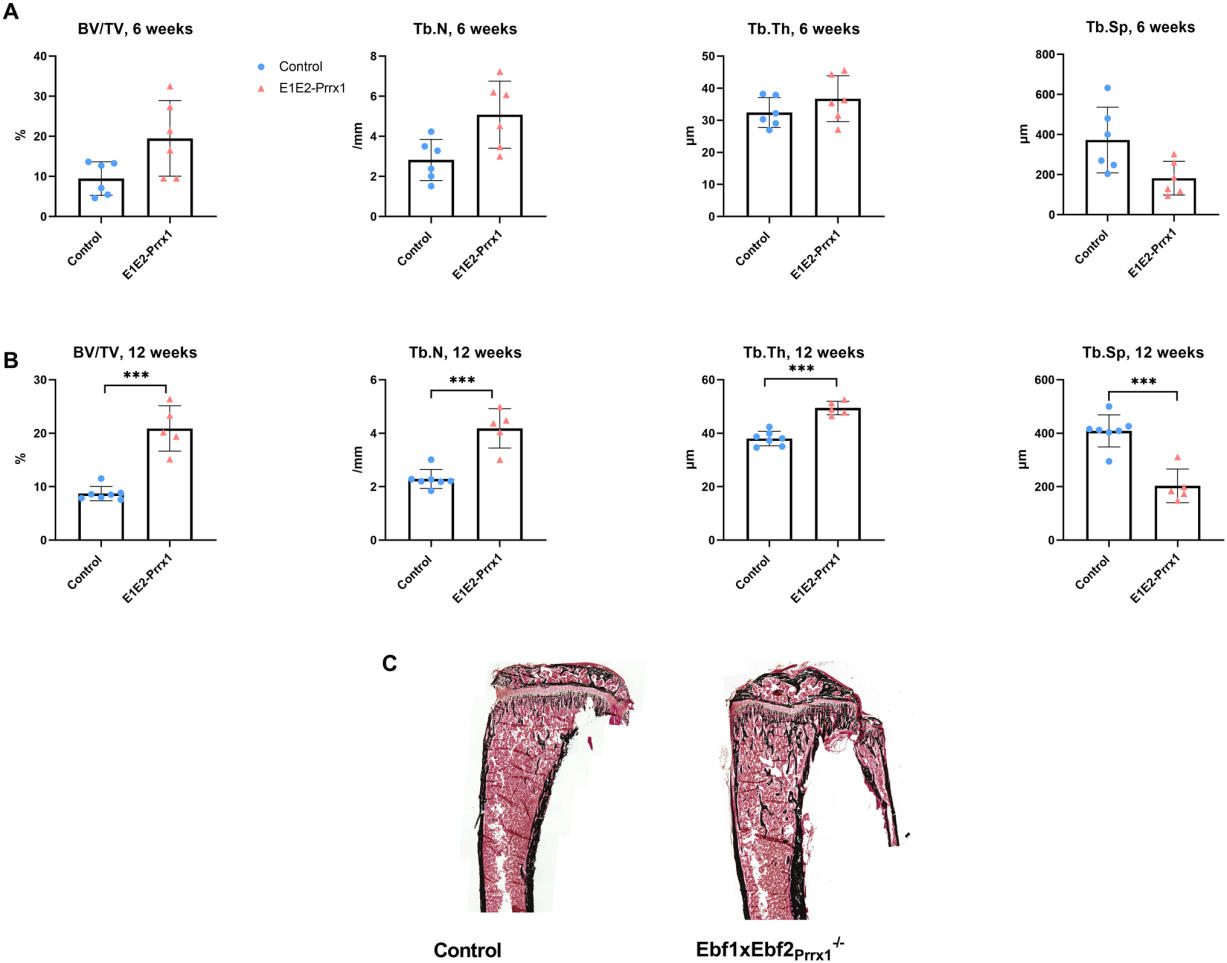
Structural histomorphometry of 6- and 12-week-old Ebf1xEbf2_Prrx1_ female mice tibia. At 6 weeks Ebf1xEbf2_Prrx1_^−/−^ (*n* = 6) structural parameters were comparable to controls (*n* = 6) (**A**). At 12 weeks the structural parameters were significantly different between Ebf1xEbf2_Prrx1_^−/−^ (*n* = 5) and controls (*n* = 7) (**B**). Representative images of Von Kossa-stained tibial sections of 12-week-old control and Ebf1xEbf2_Prrx1_^−/−^ (**C**). Statistical significance was tested by two-tailed Student’s *t*-test. *P*-values for significant differences between genotypes are presented. ***P < 0.001

Results of the histomorphometry analysis of the female Ebf1xEbf2_Prrx1_^−/−^ tibia were in accordance with the tibia μCT-results. At 6 weeks of age, results were comparable to controls (Fig. 6A), but at 12 weeks structural parameters (BV/TV, Tb.Th, Tb.N) were significantly increased compared to controls (Fig. 6B). Dynamic parameters (mineral apposition rate, MAR; bone formation rate, BFR) were unchanged in both age groups (Supplemental table 3). At 12 weeks of age, osteoclast number (N.Oc/T.Ar) was significantly increased in Ebf1xEbf2_Prrx1_^−/−^ females (*P* < 0.001) compared to controls (Supplemental table 3).

To get insight into the possible mechanisms underlying the increased bone mass observed in the Ebf1xEbf2_Prrx1_^−/−^ females, mRNA expression of selected bone-specific genes was analysed in controls and Ebf1xEbf2_Prrx1_^−/−^, at 6 and 12 weeks, in males and in females. Gene expression data did not reveal any statistically significant differences in the expression levels of Runx2, Col1a, ALP or Osx between Ebf1xEbf2_Prrx1_^−/−^ and controls, in males or females, at 6 weeks (Supplemental Fig. 5a) or at 12 weeks (Supplemental Fig. 5b). We also analysed Ebf1 and Ebf2 expression in total bone RNA samples, in both 6 and 12 week old mice, in both genders. The expression pattern in bone does not fully reflect the conditional deletion of Ebf1 and Ebf2 in mesenchymal cell lineage, as Ebfs are also expressed in other cell types of the heterogenic bone sample. Observed increase (ns, *P* = 0.07) in Ebf2 expression in six-week-old Ebf1xEbf2_Prrx1_^−/−^ could indicate a possible compensation due to Ebf1 deletion. (Supplemental Fig. 6).

### Abnormalities in the Growth Plate and Secondary Ossification in Ebf1xEbf2_Prrx1_ Males

**Fig. 7:**
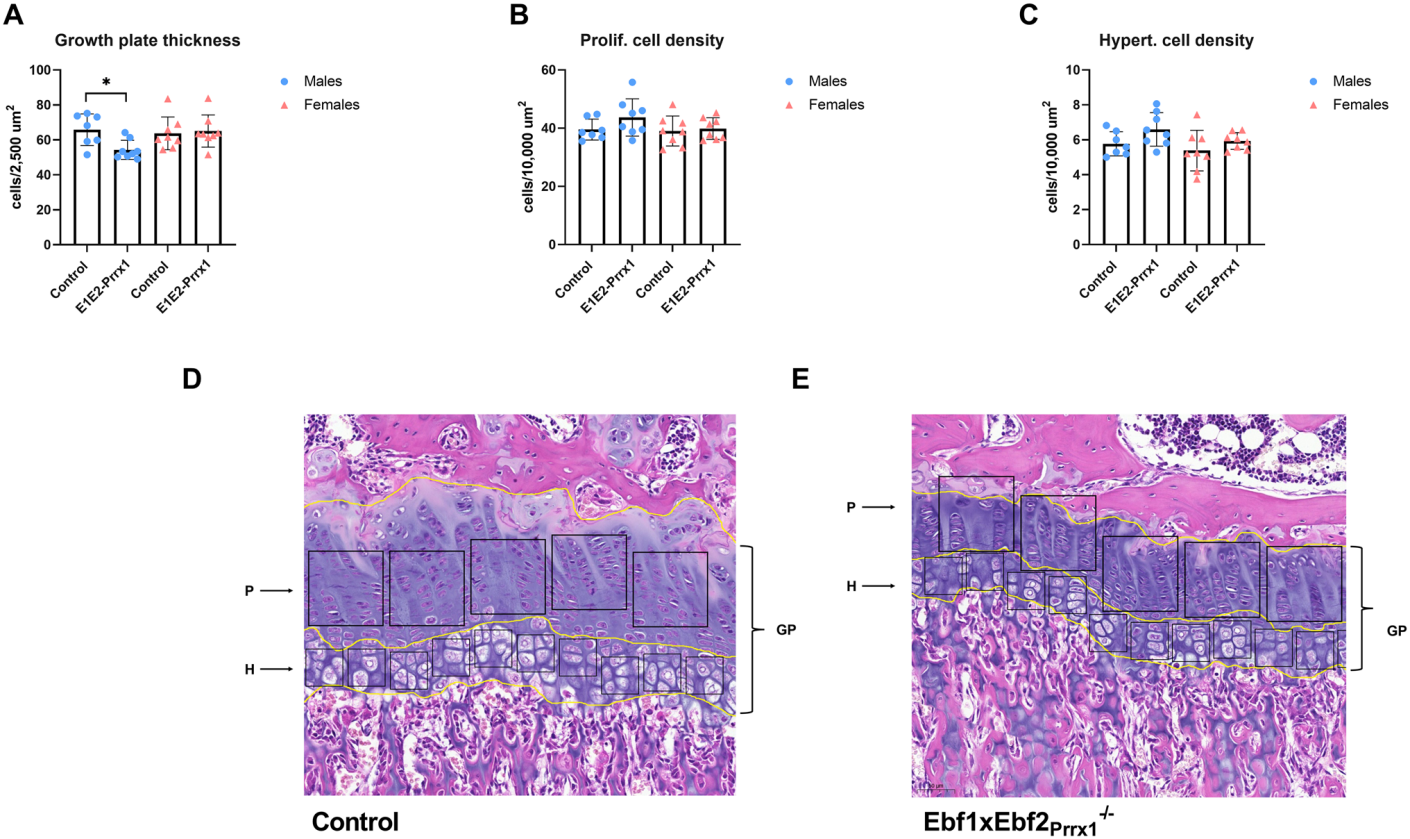
Tibial growth plate analysis of the six-week-old Ebf1xEbf2_Prrx1_ mice. Growth plate thickness was significantly reduced in Ebf1xEbf2_Prrx1_^−/−^ male mice (*n* = 8) compared to controls (*n* = 7). Female mice (*n* = 8) were comparable to controls (*n* = 8) (**A**). Proliferative zone (**B**) and hypertrophic zone (**C**) cellular densities were comparable to controls in both genders. Representative images on the analysis site of growth plate (GP) thickness and proliferative (P) and hypertrophic (H) zones in control male (**D**) and Ebf1xEbf2_Prrx1_^−/−^ male (**E**). Statistical significance was tested by two-tailed Student’s *t*-test. *P*-values for significant differences between genotypes are presented. *P < 0.05

Decreased bone length in Ebf1xEbf2_Prrx1_^−/−^ mice encouraged us to analyse the growth plate of the six-week-old Ebf1xEbf2_Prrx1_ mice. In Ebf1xEbf2_Prrx1_^−/−^ males, the thickness of the growth plate was significantly reduced (*P* = 0.02) (Fig. 7A). There were no differences in the cellular density of the proliferative zone (*P* = 0.14) or the hypertrophic zone (*P* = 0.08) (Fig. 7B, C). No significant differences were observed in female mice in any of the growth plate parameters studied (Fig. 7A–C), despite the significantly shorter bones in Ebf1xEbf2_Prrx1_^−/−^ females (Fig. 3B, D).

Due to the changes in the bone length and growth plate thickness, we further analysed the area of secondary ossification centre (SOC) in Ebf1xEbf2_Prrx1_ tibias by μCT. Trabecular number was reduced (*P* = 0.049) in the SOC of Ebf1xEbf2_Prrx1_^−/−^ males at 6 weeks (Supplemental Table 4), but at 12 weeks the bone parameters in both males and females were indistinguishable from the controls (Supplemental Table 4).

To get further insight into the role of Ebfs in secondary ossification , we also analyzed the SOC of Ebf2_Prrx1_ and previously published Ebf1_Osx_ [13] mice in more detail. There were no significant changes in the endochondral ossification in Ebf2_Prrx1_^−/−^ or Ebf1_Osx_^−/−^ mice, in males or females (Supplemental Table 4).

We also analysed the amount of bone marrow adipocytes in the SOC area and in the bone marrow cavity. Ebf1xEbf2_Prrx1_^−/−^ males had significantly more adipocytes (*P* = 0.001) in the SOC than the control mice at the age of 6 weeks (Supplemental Fig. 7c). By 12 weeks of age, the adipocyte numbers were comparable to controls (*P* > 0.05) (Supplemental Fig. 7d). There were no differences between the genotypes in the number of adipocytes in the bone marrow cavity (Supplemental Fig. 7e) in the males. No differences were observed in the SOC adiposity of the females (*P* > 0.05) (Supplemental Fig. 7f).

## Discussion

Ebfs are known to play a role in bone formation. Global deletion of Ebf1 leads to runted stature and increased bone formation [11]. We and others have previously shown that conditional deletion of Ebf1 during early stages of osteoblast differentiation leads to increased bone formation [10, 13]. Global Ebf2 deletion, in turn, has previously been shown to result in dwarfism and impaired viability in mice [2, 14]. Global Ebf2KO mice have reduced bone mass and increased amount of osteoclasts due to osteoblast-autonomous decrease in the expression of OPG [14]. The phenotype of our global Ebf2KO mice was in line with previous studies showing reduced size, body weight and bone parameters. As Ebf2 is also expressed in other cell types than bone, such as neuronal cells, bone phenotype in the Ebf2KO mice may, at least in part, be secondary to extra-skeletal effects.

To target the effect of Ebf2 deletion more specifically to bone, we created a limb bud mesenchymal cell specific Ebf2 knockout mouse model using Prrx1-promoter. Interestingly, Ebf2_Prrx1_^−/−^ mice had normal body weight and length of long bones when compared to the controls. The overall bone phenotype in the Ebf2_Prrx1_^−/−^ mice was mild. This may indicate that the bone phenotype observed in the global Ebf2KO is of non-mesenchymal cell origin.

Previous studies have suggested a possible co-operation between different Ebfs on bone formation [9, 13]. To study this in more detail, we created a conditional double knockout mouse model by deleting both Ebf1 and Ebf2 under the mesenchymal cell specific Prrx1-promoter. The focus of our study was to analyse the postnatal effects of Ebf1 and Ebf2 deletion in long bone development. Analysis of proximal and distal bones isolated from the limbs of Ebf1xEbf2_Prrx1_^−/−^ mice revealed a significant, age progressive decrease in length of long bones when compared to controls. Given the central role of chondrocyte proliferation and differentiation in the longitudinal bone growth, we analysed the growth plates from histological sections of six-week-old Ebf1xEbf2_Prrx1_ mouse tibias. The overall thickness of the growth plate was significantly decreased in the Ebf1xEbf2_Prrx1_^−/−^ male mice when compared to the controls. In females, there were no statistically significant differences in the growth plate, even though the bones in females were also significantly shorter when compared to controls.

In previously published global knockout models, the bone length was significantly reduced in both Ebf1KO [11] and Ebf2KO [14] mice. However, thickness of the growth plate was decreased only in Ebf2KO. In conditional knockout models using Runx2 or Prrx1 promoters for the conditional deletion of Ebf1 [9, 12, 13], changes in bone length have not been reported. Our conditional Ebf2 knockout model, Ebf2_Prrx1_^−/−^_,_ had normal bone length when compared to controls. Collectively, these findings indicate that the bone length defect seen in the global knockout models of either Ebf1 or Ebf2 alone arises from cells of non-mesenchymal origin. In terms of bone-specific knockout models, complete ablation of both Ebf1 and Ebf2 in mesenchymal lineage is necessary to perturb growth plate maturation and growth of long bones.

We have previously shown that Ebf1 promotes early osteoblast differentiation by regulating Osterix expression [13]. Osterix is also expressed in chondrocytes [18], and it regulates bone growth through chondrocyte hypertrophy [19]. Xing et al. recently showed that deletion of Osterix specifically in the chondrocytes impairs secondary ossification [20]. It is therefore possible that the defect in the length of Ebf1xEbf2_Prrx1_^−/−^ mouse limbs could be linked to the regulation of hypertrophic chondrocytes by Osterix.

Skeletal elements formed via endochondral ossification are derived from cartilaginous templates that are replaced by bone matrix produced by osteoblasts [21]. Decrease in bone mass is therefore frequently observed in mice with mutations that affect the formation of the cartilaginous template by altering chondrocyte biology [22, 23]. However, in this study, μCT analysis of the Ebf1xEbf2_Prrx1_ long bones revealed an increase in bone volume. Ebf1xEbf2_Prrx1_^−/−^ female mice had age progressive increase in trabecular bone and cortical bone parameters. Histomorphometric analysis further verified an increase in structural bone parameters, while dynamic parameters remained unaffected. Based on these findings, Ebf1xEbf2_Prrx1_^−/−^ mice appear to have an uncoupled bone phenotype, with shorter bones but increased trabecular and cortical bone volume in the long bones of the females.

Seike et al. recently reported a role for Ebf1 and Ebf3 in the maintenance of the bone marrow cavity [9]. They reported no gross bone abnormalities when Ebf1 was deleted in Prrx1 expressing cells, whereas Ebf3 deletion presented age progressive occlusion of bone marrow cavities. The occlusion was further enhanced when both Ebf1 and Ebf3 were deleted. However, targeting Ebf1 and Ebf3 deletion in the osteoblast lineage using Col2.3 promoter had no effect on bone phenotype.[9] These results are in line with our previous findings [13], indicating Ebf1 to be redundant in the maintenance of mature osteoblast function. Our data is also in accordance with a more recently published mouse model of Prrx1-Cre targeted deletion of Ebf1 in mesenchymal cell-lineage, where trabecular bone volume and osteoblast numbers were modestly but significantly increased [10].

Molecular mechanisms behind the bone phenotype of conditional Ebf1xEbf2_Prrx1_ double knockout remain to be further elucidated. It is known that the members of the Ebf family have a unique and structurally highly similar DNA-binding domain (DBD) [25]. Further analysis has shown the DBD to act as a dimer [24]. Based on the DBD structure and transcription factor immunoglobulin (TIG) domain, Ebfs have been suggested to resemble the Rel superfamily of transcription factors [24, 25]. This might give us clues to the possible co-operative and compensatory roles of Ebfs. Due to the structural similarity, it might be possible that certain functions require the simultaneous presence or absence of different Ebfs in gene regulation. Consequently, single deletions of Ebf1, Ebf2 of Ebf3 in bone cells result in different bone phenotypes than double knockout combinations.

Previously no gender specific bone phenotypes have been reported in Ebf1, Ebf2 or Ebf3 knockout mouse models However, previous reports [9, 11, 14] have not specified whether the data presented is obtained from male or female mice, or if both genders have been analysed. We are the first to present a sexually dimorphic bone phenotype in mice lacking both Ebf1 and Ebf2. Skeletal dimorphism in mice is known to be established during early puberty, around 3 to 5 weeks of age, and the rate of this early growth spurt is significantly greater in male mice [26]. Previous studies have indicated this early puberty to be a critical developmental period for the development of sexually dimorphic outcome in endochondral ossification [27, 28]. Female mice have also been shown to have more osteoblasts [29]. Temporal and gender specific patterns in the development and differentiation of bone cells might therefore partially explain the sexually dimorphic bone phenotype observed in Ebf1xEbf2_Prrx1_ mice.

This study has some limitations. The number of mice obtained at each time point varied, and at the age of 12-weeks the study groups of Ebf2_Prrx1_ male mice were unbalanced, resulting in only three mice in the control group and 11 in the Ebf2_Prrx1_^−/−^ group. Further, only male mice were analysed from the global Ebf2KO study. Analysis of osteoblasts isolated from control and knockout mouse lines and differentiated in vitro could provide us further information on the possible mechanisms underlying the high bone mass phenotype in Ebf1xEbf2_Prrx1_ mice. Finally, with our study design we were not able to determine the detailed mechanism for gender specific differences.

In summary, we found that the conditional deletion of Ebf1 and Ebf2 in mesenchymal lineage cells leads to a significant, age progressive bone phenotype. This phenotype is to some extent gender dependent, leading to an increase in both trabecular and cortical bone in females. In males, in contrast, the absence of both Ebf1 and Ebf2 resulted in a mild cortical bone phenotype and a growth plate defect. Further studies on the molecular mechanisms and on the possible role of Ebf3 are still needed to elucidate the role of Ebfs in bone cells.

## Supplementary Information

Below is the link to the electronic supplementary material.

Supplementary file1 (PDF 873 kb)

## References

[1] Wang SS, Lewcock JW, Feinstein P, Mombaerts P, Reed RR (2004). Genetic disruptions of O/E2 and O/E3 genes reveal involvement in olfactory receptor neuron projection. Development.

[2] Corradi A, Croci L, Broccoli V, Zecchini S, Previtali S, Wurst W, Amadio S, Maggi R, Quattrini A, Consalez GG (2003). Hypogonadotropic hypogonadism and peripheral neuropathy in Ebf2-null mice. Development.

[3] Hagman J, Belanger C, Travis A, Turck CW, Grosschedl R (1993). Cloning and functional characterization of early B-cell factor, a regulator of lymphocyte-specific gene expression. Genes Dev.

[4] Lin YC, Jhunjhunwala S, Benner C, Heinz S, Welinder E, Mansson R, Sigvardsson M, Hagman J, Espinoza CA, Dutkowski J, Ideker T, Glass CK, Murre C (2010). A global network of transcription factors, involving E2A, EBF1 and Foxo1, that orchestrates B cell fate. Nat Immunol.

[5] Akerblad P, Månsson R, Lagergren A, Westerlund S, Basta B, Lind U, Thelin A, Gisler R, Liberg D, Nelander S, Bamberg K, Sigvardsson M (2005). Gene expression analysis suggests that EBF-1 and PPARgamma2 induce adipogenesis of NIH-3T3 cells with similar efficiency and kinetics. Physiol Genomics.

[6] Jimenez MA, Åkerblad P, Sigvardsson M, Rosen ED, Akerblad P, Sigvardsson M, Rosen ED (2007). Critical role for Ebf1 and Ebf2 in the adipogenic transcriptional cascade. Mol Cell Biol.

[7] Griffin MJ, Zhou Y, Kang S, Zhang X, Mikkelsen TS, Rosen ED (2013). Early B-cell factor-1 (EBF1) is a key regulator of metabolic and inflammatory signaling pathways in mature adipocytes. J Biol Chem.

[8] Rajakumari S, Wu J, Ishibashi J, Lim HW, Giang AH, Won KJ, Reed RR, Seale P (2013). EBF2 determines and maintains brown adipocyte identity. Cell Metab.

[9] Seike M, Omatsu Y, Watanabe H, Kondoh G, Nagasawa T (2018). Stem cell niche-specific Ebf3 maintains the bone marrow cavity. Genes Dev.

[10] Derecka M, Herman JS, Cauchy P, Ramamoorthy S, Lupar E, Grün D, Grosschedl R (2020). EBF1-deficient bone marrow stroma elicits persistent changes in HSC potential. Nat Immunol.

[11] Hesslein DGT, Fretz JA, Xi Y, Nelson T, Zhou S, Lorenzo JA, Schatz DG, Horowitz MC (2009). Ebf1-dependent control of the osteoblast and adipocyte lineages. Bone.

[12] Zee T, Boller S, Györy I, Makinistoglu MP, Tuckermann JP, Grosschedl R, Karsenty G (2013). The transcription factor early B-cell factor 1 regulates bone formation in an osteoblast-nonautonomous manner. FEBS Lett.

[13] Nieminen-Pihala V, Tarkkonen K, Laine J, Rummukainen P, Saastamoinen L, Nagano K, Baron R, Kiviranta R (2021). Early B-cell factor1 (Ebf1) promotes early osteoblast differentiation but suppresses osteoblast function. Bone.

[14] Kieslinger M, Folberth S, Dobreva G, Dorn T, Croci L, Erben R, Consalez GG, Grosschedl R (2005). EBF2 regulates osteoblast-dependent differentiation of osteoclasts. Dev Cell.

[15] Liu D, Lin Z, Huang Y, Qiu M (2021). Role of microRNA-19b-3p on osteoporosis after experimental spinal cord injury in rats. Arch Biochem Biophys.

[16] Logan M, Martin JF, Nagy A, Lobe C, Olson EN, Tabin CJ (2002). Expression of Cre recombinase in the developing mouse limb bud driven by a Prxl enhancer. Genesis.

[17] Dempster DW, Compston JE, Drezner MK, Glorieux FH, Kanis JA, Malluche H, Meunier PJ, Ott SM, Recker RR, Parfitt AM (2013). Standardized nomenclature, symbols, and units for bone histomorphometry: a 2012 update of the report of the ASBMR Histomorphometry Nomenclature Committee. J Bone Miner Res.

[18] Nishimura R, Wakabayashi M, Hata K, Matsubara T, Honma S, Wakisaka S, Kiyonari H, Yamaguchi A, Tsumaki N, Akiyama H, Yoneda T (2012). Osterix regulates calcification and degradation of chondrogenic matrices through matrix metalloproteinase 13 (MMP13) Expression in association with transcription factor Runx2 during endochondral ossification * S. J Biol Chem.

[19] Cheng S, Xing W, Zhou X, Mohan S (2013). Haploinsufficiency of osterix in chondrocytes impairs skeletal growth in mice. Physiol Genomics.

[20] Xing W, Godwin C, Pourteymoor S, Mohan S (2019). Conditional disruption of the osterix gene in chondrocytes during early postnatal growth impairs secondary ossification in the mouse tibial epiphysis. Bone Res.

[21] Provot S, Schipani E (2005). Molecular mechanisms of endochondral bone development. Biochem Biophys Res Commun.

[22] Maeda Y, Nakamura E, Nguyen MT, Suva LJ, Swain FL, Razzaque MS, Mackem S, Lanske B (2007). Indian Hedgehog produced by postnatal chondrocytes is essential for maintaining a growth plate and trabecular bone. Proc Natl Acad Sci USA.

[23] Tang J, Xie J, Chen W, Tang C, Wu J, Wang Y, Zhou XD, De Zhou H, Li YP (2020). Runt-related transcription factor 1 is required for murine osteoblast differentiation and bone formation. J Biol Chem.

[24] Treiber N, Treiber T, Zocher G, Grosschedl R (2010). Structure of an Ebf1:DNA complex reveals unusual DNA recognition and structural homology with Rel proteins. Genes Dev.

[25] Siponen MI, Wisniewska M, Lehtiö L, Johansson I, Svensson L, Raszewski G, Nilsson L, Sigvardsson M, Berglund H (2010). Structural determination of functional domains in early B-cell factor (EBF) family of transcription factors reveals similarities to rel DNA-binding proteins and a novel dimerization motif. J Biol Chem.

[26] Callewaert F, Venken K, Kopchick JJ, Torcasio A, Van Lenthe GH, Boonen S, Vanderschueren D (2010). Sexual dimorphism in cortical bone size and strength but not density is determined by independent and time-specific actions of sex steroids and IGF-1: evidence from pubertal mouse models. J Bone Miner Res.

[27] Linz A, Knieper Y, Gronau T, Hansen U, Aszodi A, Garbi N, Hämmerling GJ, Pap T, Bruckner P, Dreier R (2015). ER stress during the pubertal growth spurt results in impaired long-bone growth in chondrocyte-specific ERp57 knockout mice. J Bone Miner Res.

[28] Fairfield H, Costa S, DeMambro V, Schott C, Martins JDS, Ferron M, Vary C, Reagan MR (2020). Targeting bone cells during sexual maturation reveals sexually dimorphic regulation of endochondral ossification. JBMR Plus.

[29] Zanotti S, Kalajzic I, Aguila HL, Canalis E (2014). Sex and genetic factors determine osteoblastic differentiation potential of murine bone marrow stromal cells. PLoS ONE.

