# Supplementary material for: Age-Progressive and Gender-Dependent Bone Phenotype in Mice Lacking Both Ebf1 and Ebf2 in Prrx1-Expressing Mesenchymal Cells
Source: Calcif Tissue Int. 2022 Feb 8;110(6):746–58. doi: 10.1007/s00223-022-00951-7 (PMC9108109; doi:10.1007/s00223-022-00951-7)
Supplement: Supplementary file 1 — Supplementary file1 (PDF 873 kb) [file 223_2022_951_MOESM1_ESM.pdf]

Supplementary material

Age-progressive and gender-dependent bone phenotype in mice lacking both Ebf1 and Ebf2 in the Prrx1-expressing mesenchymal cells

Vappu Nieminen-Pihala[a*], Petri Rummukainen[a*], Fan Wang[a], Kati Tarkkonen[a,b], Kaisa K. Ivaska[a] and Riku Kiviranta[a,c,]

[a] Institute of Biomedicine, University of Turku, Turku, Finland

[b] Present address: Orion Pharma, Turku, Finland

[c] Department of Endocrinology, Division of Medicine, University of Turku and Turku University Hospital, Turku, Finland

* These authors contributed equally to this paper

Corresponding author: Vappu Nieminen-Pihala, vamani@utu.fi

Supplemental figure S1.

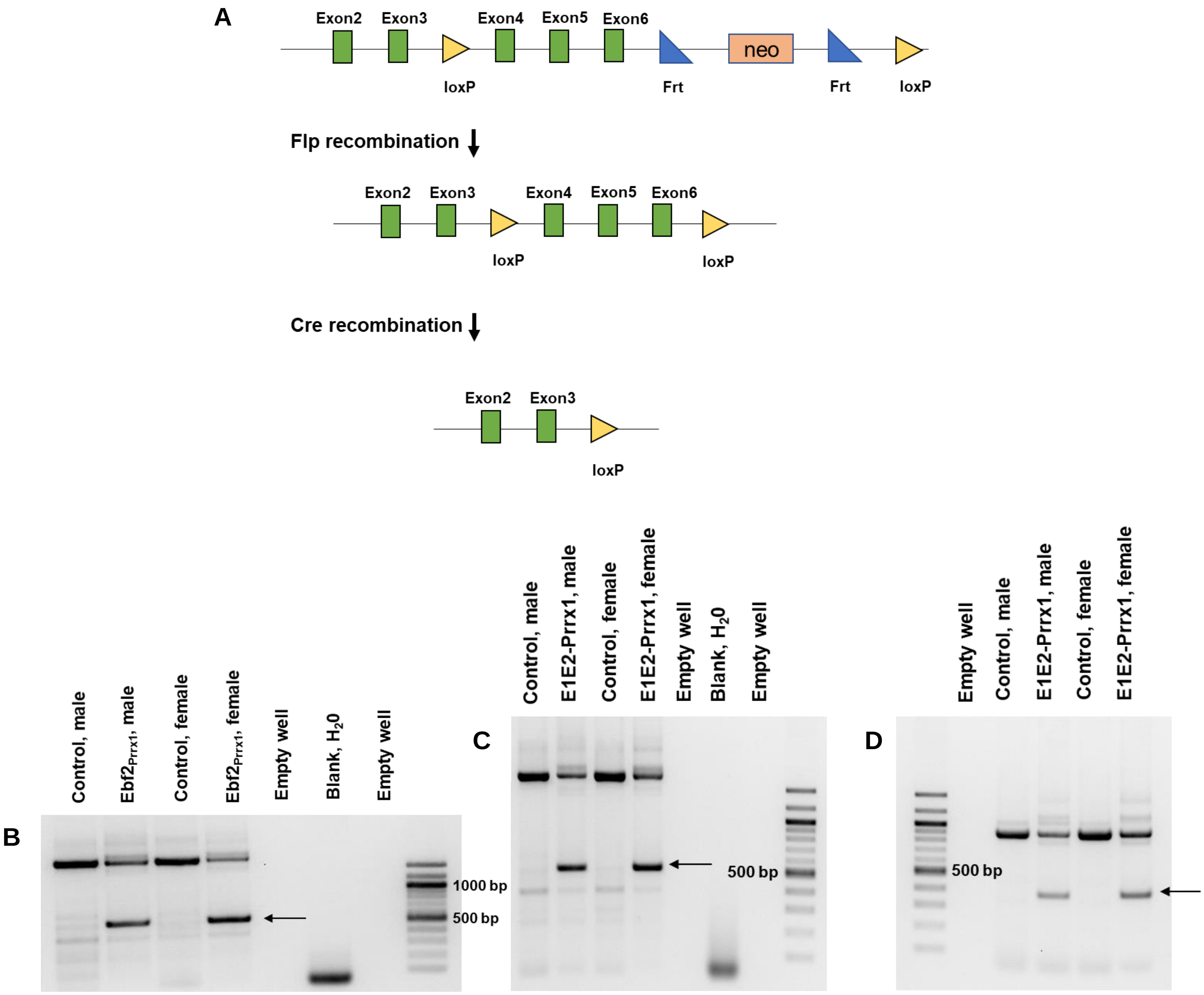

Fig. S1 Schematic presentation of the generation of conditional Ebf2 knockout mice (**a**). Verification of the Ebf2 deletion from $Ebf2_{Prrx1}$ DNA samples extracted from humeri. The primers were designed to amplify Ebf2 deletion band (496 bp, black arrow) after the Cre recombination has deleted the exons 4-6. (**b**) Verification of the Ebf1 and Ebf2 deletion from $Ebf1xEbf2_{Prrx1}$ DNA samples extracted from humeri. The primers were designed to amplify Ebf2 deletion band (496 bp, black arrow) (**c**). A second pair of primers was designed to amplify Ebf1 deletion band (392 bp, black arrow) after the Cre recombination has deleted the exon three (**d**). The deletion pattern was similar in males and females.

Supplemental figure S2.

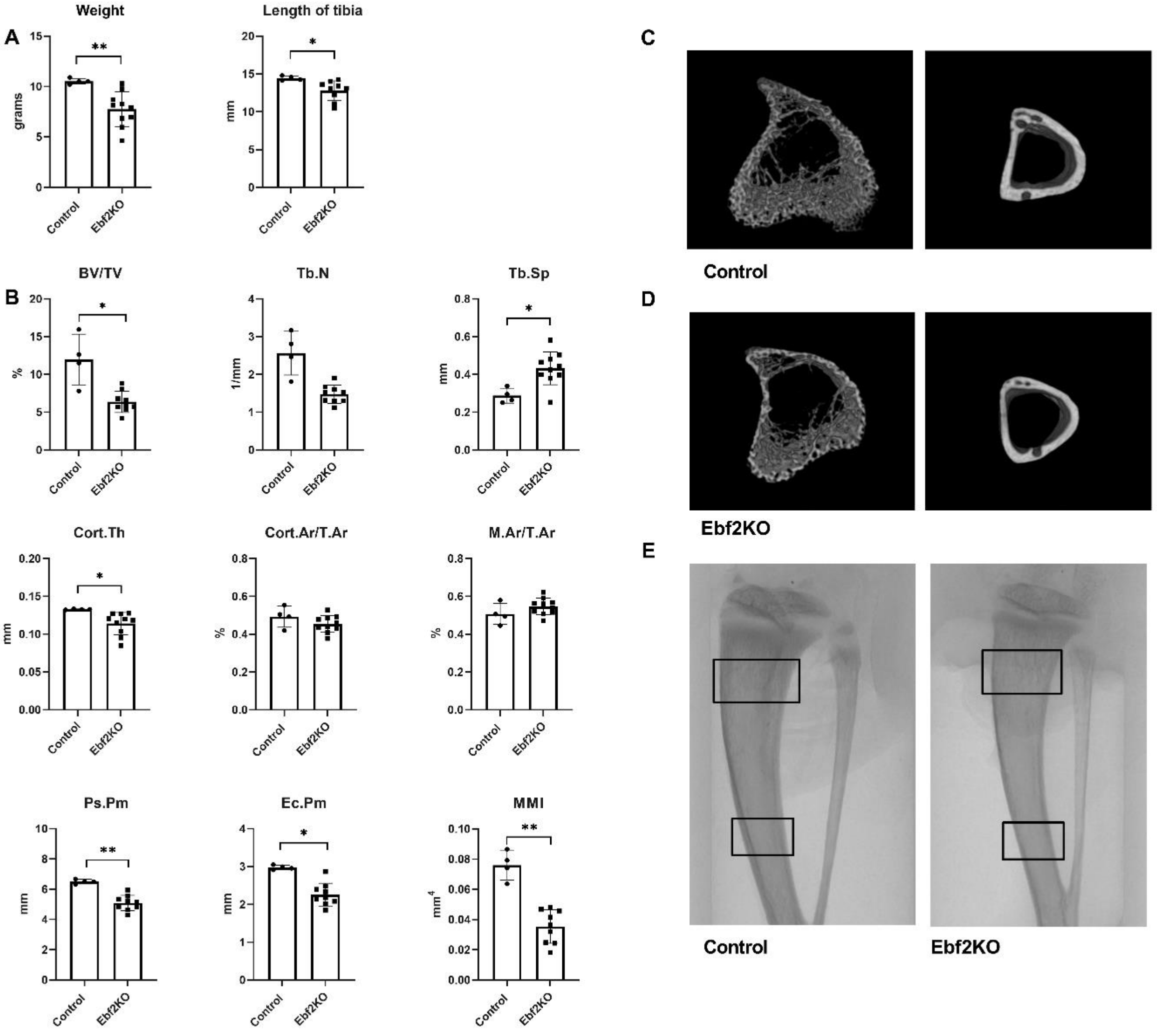

Fig. S2 Bone phenotype of the three-week-old Ebf2KO male mice. Global deletion of Ebf2 led to significant reduction in body weight and length of tibia in knockout mice (n=10) compared to controls (n=4) (**a**). Tibial µCT-analysis showed significantly reduced trabecular and cortical bone parameters in the Efb2KO male mice when compared to controls (**b**). 3D rendered representation of the trabecular and cortical bone region of interest in control mouse (**c**) and Ebf2KO mouse (**d**). Representative coronal view of the tibia, trabecular ROI and cortical ROI are marked with black squares (**e**). Statistical significance was tested by two-tailed Student's t-test with Bonferroni correction. * $P < 0.05$; **, $P < 0.01$

Supplemental figure S3.

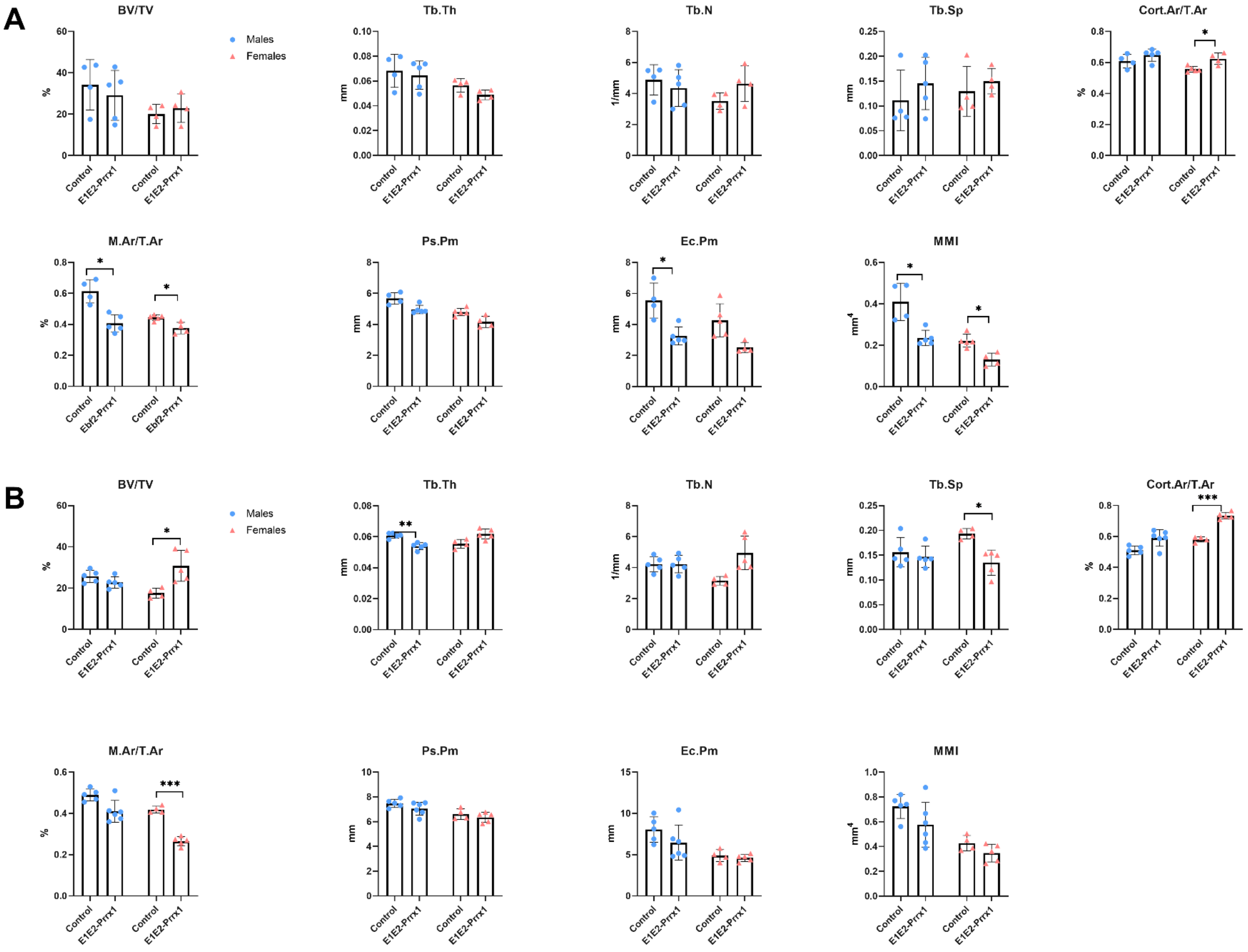

Fig. S3 µCT-analysis of 6-week-old and 12-week-old Ebf1xEbf2$_{Prrx1}$ mice tibia. At six weeks marrow area fraction, endocortical perimeter and mean moment of inertia were decreased in Ebf1xEbf2$_{Prrx1}^{-/-}$ (n=5) males compared to controls (n=4). Ebf1xEbf2$_{Prrx1}^{-/-}$ females (n=4) had significantly increased cortical bone fraction compared to controls (n=5). (**a**) At 12 weeks trabecular bone thickness was significantly increased in Ebf1xEbf2$_{Prrx1}^{-/-}$ males (n=5) compared to controls (n=5). In Ebf1xEbf2$_{Prrx1}^{-/-}$ females (n=5) trabecular bone volume and cortical bone fraction were significantly increased compared to controls (n=4). (**b**) Statistical significance was tested by two-tailed Student's t-test with Bonferroni correction. P-values for significant differences between genotypes are presented. * P < 0.05; **, P < 0.01; ***, P < 0.001

Supplemental figure S4.

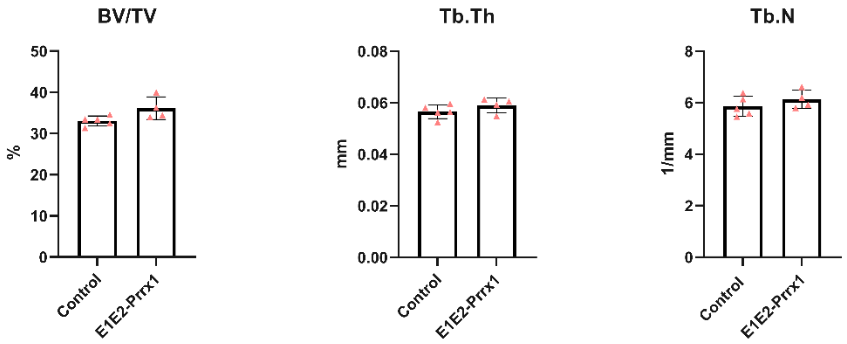

Fig. S4 µCT-analysis of 12-week-old Ebf1xEbf2$_{Prrx1}$ female mice vertebra. Trabecular bone parameters of the Ebf1xEbf2$_{Prrx1}^{-/-}$ (n=4) female vertebra were comparable to controls (n=5). Statistical significance was tested by two-tailed Student's t-test with Bonferroni correction.

Supplemental figure S5.

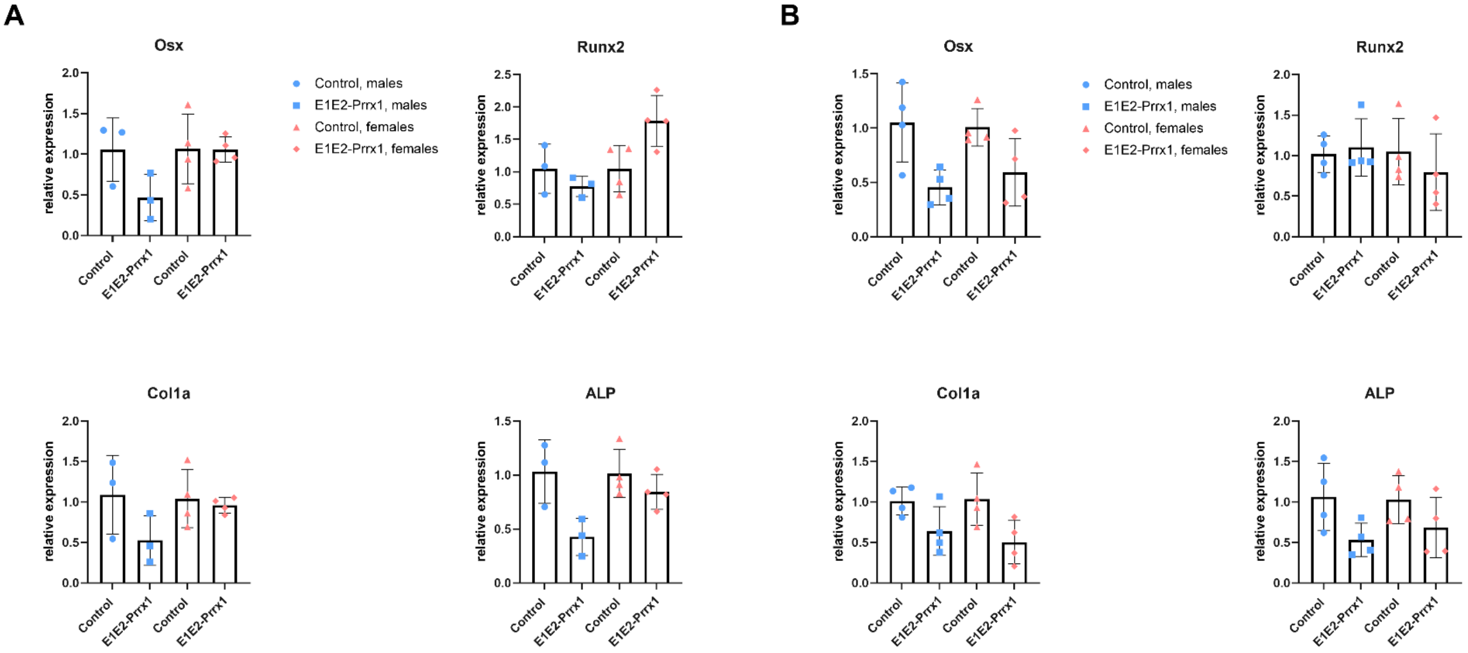

Fig. S5 Quantitative real-time PCR (qPCR) expression analysis of bone-related genes from bone RNA of 6-week-old (**a**) and 12-week-old (**b**) Ebf1xEbf2$_{Prrx1}$ mice (E1E2-Prrx1). Relative expression was normalized to the expression of beta-actin. Results were calculated using deltadelta Ct method and controls were set as 1. In 6-week samples n=3 males and n=4 females both in control and Ebf1xEbf2$_{Prrx1}$$^{-/-}$. In 12-week samples n=4 males and n=4 in females both in control and Ebf1xEbf2$_{Prrx1}$$^{-/-}$. Statistical significance was tested by two-tailed Student’s t-test and Benjamini-Hochberg Procedure.

Supplemental figure S6.

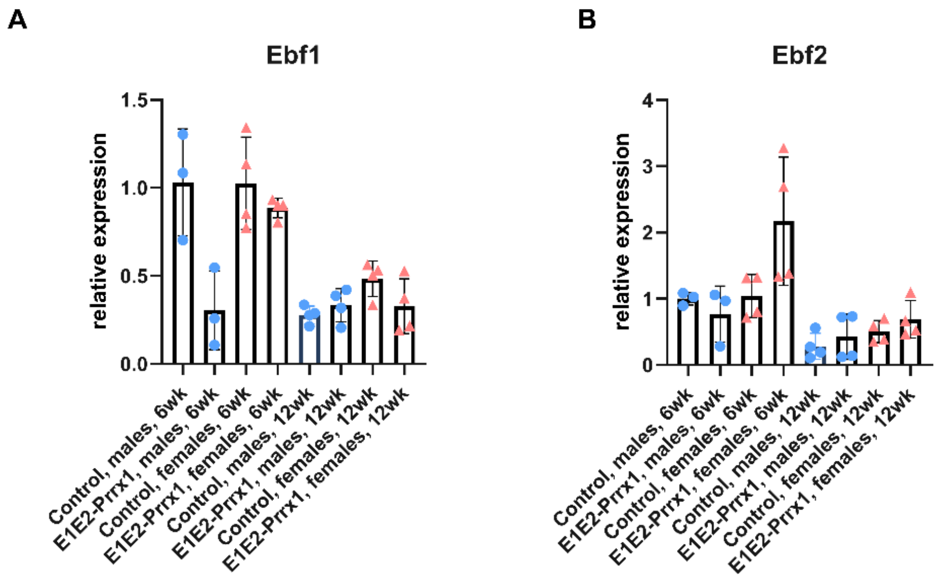

Fig. S6 Quantitative real-time PCR (qPCR) expression analysis of Ebf1 (**a**) and Ebf2 (**b**) from bone RNA of 6-week-old and 12-week-old Ebf1xEbf2$_{Prrx1}$ mice (E1E2-Prrx1). Relative expression was normalized to the expression of beta-actin. Results were calculated using deltadelta Ct method and controls were set as 1. In 6-week samples n=3 males and n=4 females both in control and Ebf1xEbf2$_{Prrx1}^{-/-}$. In 12-week samples n=4 males and n=4 in females both in control and Ebf1xEbf2$_{Prrx1}^{-/-}$. Statistical significance was tested by two-tailed Student's t-test.

Supplemental figure S7.

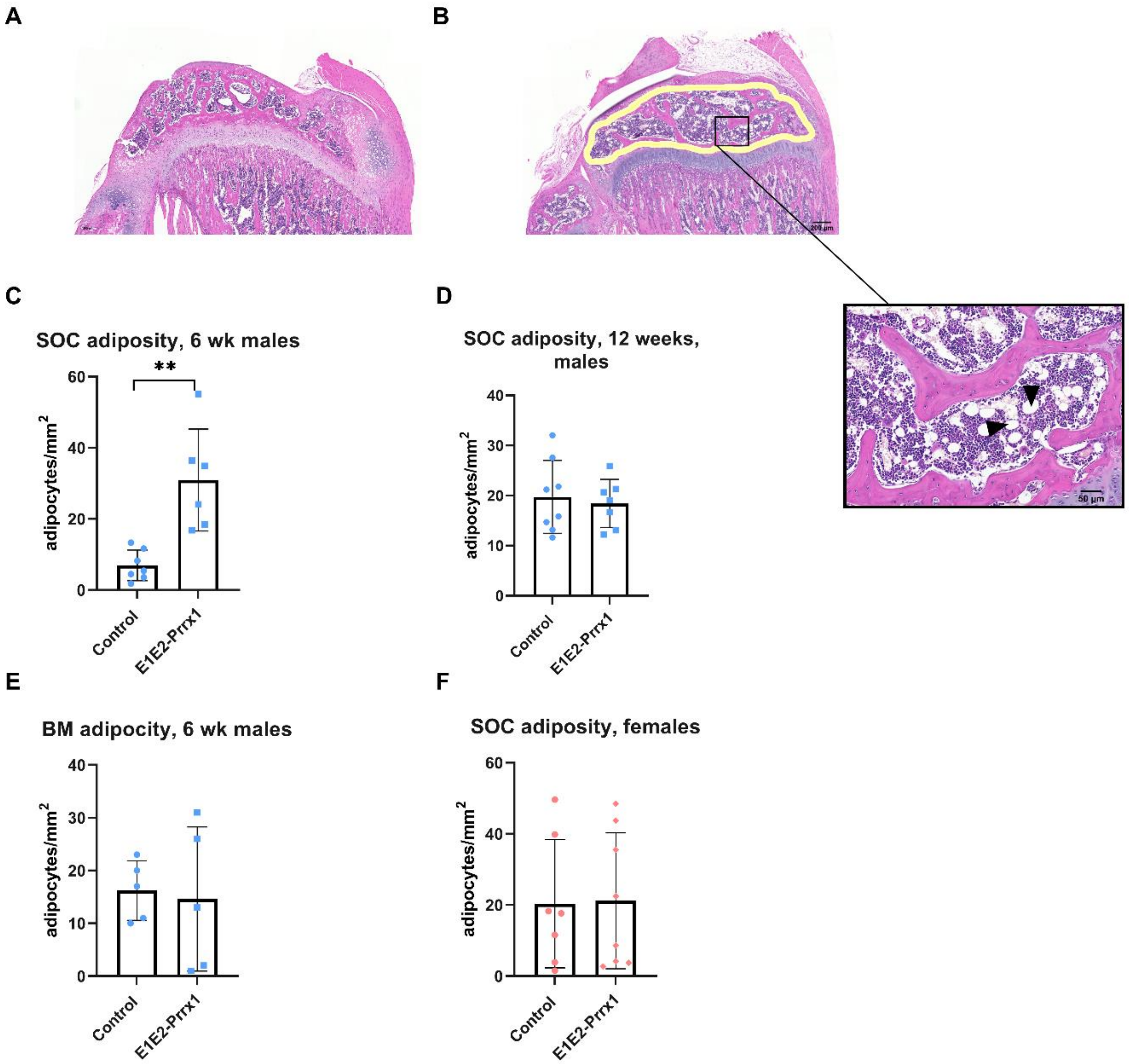

Fig. S7 Adiposity in the secondary ossification centres (SOC) and bone marrow (BM) in 6-week-old Ebf1xEbf2$_{Prrx1}$ mice. Representative images of histological sections from tibial SOC area, control male (**a**) and Ebf1xEbf2$_{Prrx1}^{-/-}$ male (**b**). Analyzed SOC area is outlined with a yellow line. Examples of two manually counted adipocyte ghosts are indicated with black arrowheads at higher magnification (**b**). SOC adiposity was significantly increased in Ebf1xEbf2$_{Prrx1}^{-/-}$ males (n=6) compared to controls (n=7) at 6 weeks (**c**). By 12 weeks of age, the adipocyte numbers in the SOC of Ebf1xEbf2$_{Prrx1}^{-/-}$ males (n=8) were comparable to controls (n=7) (**d**) Bone marrow adiposity was unaffected in the six-week-old males (n=5 control, n=5 Ebf1xEbf2$_{Prrx1}^{-/-}$) (**e**). SOC area adiposity in Ebf1xEbf2$_{Prrx1}^{-/-}$ females (n=8) was comparable to controls (n=7) (**f**)**.** Statistical significance was tested by two-tailed Student's t-test. P-values for significant differences between genotypes are presented. ** $P < 0.01$

Supplemental table 1 PCR primers used for verification of successful recombination.

Primers	Sequence	Product size after successful recombination (bp)	Used in
Ebf1del_F<br>Ebf1del_R	AACGAGCGGAACCCTACTTG<br>CTAGAGCCCTTCTGAAGCCG	392 bp	Verifying cre/loxP recombination in Ebf1cKO mouse models
Ebf2del_F<br>Ebf2del_R	CAGGGGTGTAGTTGGTTGGA<br>GCAGGGGATGAAAAGCCCAGA	496 bp	Verifying cre/loxP recombination in Ebf2cKO mouse models

Supplemental table 2 qRT-PCR primers

Target gene	Forward	Reverse
**ALP**	ACTCAGGGCAATGAGGTCAC	CACCCGAGTGGTAGTCACAA
**Col1a**	AGACATGTTCAGCTTTGTGGA	GCA GCT GAC TTCAGG GATG
**Ebf1**	AGATTGAGAGGACGGCCTTTGT	TCTGTCCGTATCCCATTGCTG
**Ebf2**	AAACCCAAGGGACATGAGG	CACATGTCCATCCACATTTACTG
**Osx**	GTCCTCTCTGCTTGAGGAAGAA	GGGCTGAAAGGTCAGCGTAT
**Runx2**	GCCCAGGCGTATTTCAGA	TGCCTGGCTCTTCTTACTGAG
**β-actin**	CGTGGGCCGCCCTAGGCACCA	TTGGCCTTAGGGTTCAGGGGG

Supplemental table 3 Dynamic histomorphometry of the 6-week-old Ebf1xEbf2$_{Prrx1}$ and 12-week-old Ebf1xEbf2$_{Prrx1}$ female mice tibia.

**6 weeks**	**Control n=6**	**Ebf1xEbf2$_{Prrx1}^{-/-}$ n=6**
**MAR (µm/day)**	2.63 ± 1.90	2.01 ± 0.79
**BFR/BV (%/day)**	1.81 ± 0.61	1.50 ± 0.66
**N.Ob/B.Pm (/mm)**	3.96 ± 1.42	3.00 ± 1.34
**Ob.S/BS (%)**	4.82 ± 1.59	3.49 ± 1.84
**N.Oc/B.Pm (/mm)**	3.74 ± 1.56	2.45 ± 0.51
**Oc.S/BS (%)**	9.02 ± 3.41	5.95 ± 1.59
**N.Oc/T.Ar (/mm^2)**	20.7 ± 9.18	25.9 ± 11.3
**12 weeks**	**Control n=6**	**Ebf1xEbf2$_{Prrx1}^{-/-}$ n=5**
**MAR (µm/day)**	1.87 ± 0.37	2.11 ± 0.46
**BFR/BV (%/day)**	2.54 ± 1.02	2.22 ± 0.66
**N.Ob/B.Pm (/mm)**	4.55 ± 3.00	2.55 ± 1.21
**Ob.S/BS (%)**	5.50 ± 3.94	2.75 ± 1.34
**N.Oc/B.Pm (/mm)**	2.90 ± 0.81	3.36 ± 0.40
**Oc.S/BS (%)**	6.62 ± 1.93	7.78 ± 1.08
**N.Oc/T.Ar (/mm^2)**	13.2 ± 4.42	27.6 ± 1.16***

Statistical significance was tested by two-tailed Student's t-test. P-values for significant differences between genotypes are presented. Statistically significant differences are indicated as *** $P < 0.001$

Supplemental table 4 µCT-analysis of the secondary ossification centre in the tibia of 6- and 12-week-old Ebf1xEbf2$_{Prrx1}$ and six-week-old Ebf2$_{Prrx1}$ mice and 12-week-old Ebf1$_{Osx}$ mice tibia.

**6-weeks**	**Males**		**Females**	
	**Control**	**Ebf1xEbf2$_{Prrx1}^{-/-}$**	**Control**	**Ebf1xEbf2$_{Prrx1}^{-/-}$**
	**n=3**	**n=4**	**n=3**	**n=4**
**Bone volume/total volume (%)**	65.8 ± 21.5	46.6 ± 12.1	52.2 ± 15.8	33.9 ± 2.13
**Trabecular thickness (mm)**	0.07 ± 0.01	0.08 ± 0.02	0.06 ± 0.01	0.06 ± 0.02
**Trabecular number (1/mm)**	9.72 ± 1.23	7.04 ± 0.64*	8.34 ± 1.12	6.07 ± 0.61
**Trabecular separation (mm)**	0.06 ± 0.02	0.08 ± 0.02	0.07 ± 0.02	0.09 ± 0.01
**6-weeks**	**Males**		**Females**	
	**Control**	**Ebf2$_{Prrx1}^{-/-}$**	**Control**	**Ebf2$_{Prrx1}^{-/-}$**
	**n=5**	**n=5**	**n=4**	**n=4**
**Bone volume/total volume (%)**	55.3 ± 10.2	52.8 ± 5.54	46.2 ± 0.66	43.9 ± 0.66
**Trabecular thickness (mm)**	0.07 ± 0.00	0.07 ± 0.01	0.07 ± 0.00	0.07 ± 0.00
**Trabecular number (1/mm)**	8.20 ± 1.25	7.44 ± 0.35	6.52 ± 0.58	6.35 ± 0.17
**Trabecular separation (mm)**	0.08 ± 0.01	0.08 ± 0.00	0.09 ± 0.00	0.09 ± 0.00
**12-weeks**	**Males**		**Females**	
	**Control**	**Ebf1xEbf2$_{Prrx1}^{-/-}$**	**Control**	**Ebf1xEbf2$_{Prrx1}^{-/-}$**
	**n=5**	**n=4**	**n=4**	**n=5**
**Bone volume/total volume (%)**	46.2 ± 3.34	37.6 ± 4.71	37.2 ± 3.30	41.6 ± 2.31
**Trabecular thickness (mm)**	0.06 ± 0.00	0.06 ± 0.00	0.06 ± 0.00	0.07 ± 0.00
**Trabecular number (1/mm)**	7.41 ± 0.53	6.08 ± 0.94	5.91 ± 0.16	6.10 ± 0.34
**12-weeks**	**Males**		**Females**	
	**Control**	**Ebf1$_{Osx}^{-/-}$**	**Control**	**Ebf1$_{Osx}^{-/-}$**
	**n=5**	**n=8**	**n=5**	**n=4**
**Bone volume/total volume (%)**	46.8 ± 6.21	41.8 ± 4.56	49.9 ± 5.52	54.3 ± 2.59
**Trabecular thickness (mm)**	0.07 ± 0.00	0.07 ± 0.00	0.08 ± 0.00	0.08 ± 0.01
**Trabecular number (1/mm)**	6.91 ± 0.63	6.11 ± 0.80	6.64 ± 0.68	7.14 ± 0.29
**Trabecular separation (mm)**	0.09 ± 0.01	0.10 ± 0.01	0.08 ± 0.00	0.07 ± 0.00

Statistical significance was tested by two-tailed Student's t-test with Bonferroni correction. P-values for significant differences between genotypes are presented. Statistically significant differences are indicated as * $P < 0.05$
